# AI-based detection of temporal changes in MR-Linac images acquired during routine prostate radiotherapy

**DOI:** 10.1016/j.phro.2026.101081

**Published:** 2026-09-23

**Authors:** Seungbin Park, Peilin Wang, Joshua Paik, Ryan Pennell, Francesca Khani, Himanshu Nagar, Timothy McClure, Emily S. Weg, Mert R. Sabuncu, Daniel Margolis, Heejong Kim

**Affiliations:** a Weill Cornell Medicine, United States of America; b The Hong Kong Polytechnic University, Hong Kong; c Memorial Sloan Kettering Cancer Center, United States of America

**Keywords:** Prostate cancer, MR-Linac, Radiation therapy, Longitudinal image analysis, Adaptive radiotherapy, Deep learning, Artificial intelligence

## Abstract

**Background and purpose:**

To investigate whether an AI-based method can detect subtle inter-fraction changes in MR-Linac images acquired during radiotherapy and explore the broader potential of MR-Linac imaging.

**Material and methods:**

This retrospective study included longitudinal 0.35T MR-Linac images from 761 patients. To identify temporal changes, we employed a deep learning model using temporal ordering via pairwise comparison, previously shown effective for longitudinal imaging studies. The model was trained using first-to-last fraction pairs ($F_{1}$-$F_{L}$) and all pairs (*All-pairs*). Performance was assessed using quantitative metrics (accuracy and AUC) and compared against a radiologist’s performance. Qualitative evaluation was performed using saliency maps, which identify anatomical regions associated with temporal imaging changes.

**Results:**

The $F_{1}$–$F_{L}$ model demonstrated high performance (AUC=0.99; accuracy=0.95) and outperformed the radiologist in temporal ordering task. The *All-pairs* model also showed high performance (AUC = 0.97; accuracy = 0.91). The performance was correlated to fractional intervals and was reduced for non-radiation-exposed timepoints (Sim and $F_{1}$). Patients who later developed biochemical recurrence exhibited smaller longitudinal changes in model logits during radiotherapy. Regions contributing to predictions included the prostate, bladder, and pubic symphysis.

**Conclusions:**

These findings demonstrate the feasibility of detecting subtle inter-fractional changes over short periods (two days on average) in routine MR-Linac images during prostate radiotherapy and contribute to the development of imaging biomarkers for treatment response.

## Highlights

• An AI model accurately predicted temporal ordering of longitudinal MR-Linac images.
• Model logits were proportional to fraction interval.
• Longitudinal model logits were associated with biochemical recurrence.
• MR-Linac imaging may serve as a potential biomarker of treatment response.

## Graphical abstract

**Figure:**
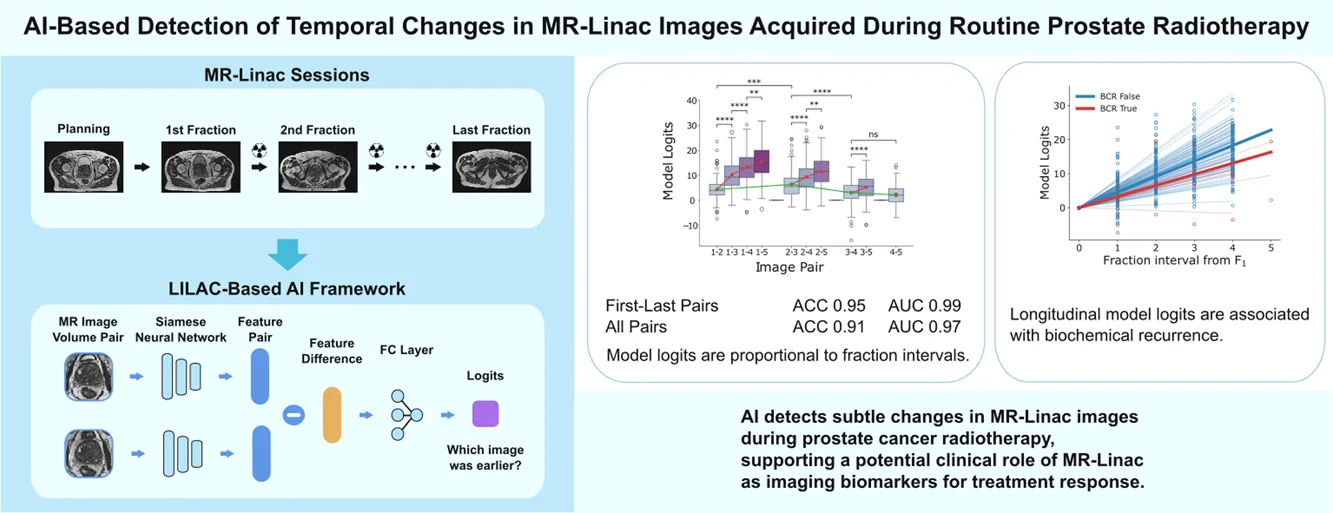

## 1 Introduction

A common therapeutic strategy for prostate cancer is external beam radiotherapy (EBRT) [1], [2]. Image-guided radiotherapy has become standard practice for ensuring precise localization of the target and safe treatment delivery [3], [4]. Magnetic resonance linear accelerator (MR-Linac) systems combine MRI with a linear accelerator and acquire MR images with high soft-tissue contrast at every fraction [3], [4], enabling visualization of the prostate, bladder, and adjacent tissues before radiation delivery and day-to-day anatomical changes throughout the treatment course [3], [4], [5]. Moreover, it allows for adaptive therapy by modifying each fraction as needed based on spatiotemporal variations to ensure maximal target dose delivery and minimal dose to adjacent organs [3], [6], [7], [8].

Several studies have investigated inter-fraction changes during prostate radiotherapy using predefined imaging biomarkers, including prostate volume [2], [9], [10], [11], multiparametric and quantitative MRI biomarkers [12], [13], [14], and apparent diffusion coefficient (ADC) measurements acquired on MR-Linac systems [15], [16]. However, these investigations have primarily focused on predefined imaging biomarkers, requiring prior assumptions on features. Whether routinely acquired MR-Linac images, with relatively low image quality and subtle changes that are challenging for human observers to detect, contain sufficient information to reveal changes throughout the course of radiotherapy without predefined biomarkers remains largely unexplored. Data-driven deep learning approaches can learn hierarchical image representations directly from the imaging data without requiring a priori specification of the relevant features [17]. Such approaches may therefore capture complex and subtle imaging patterns that are difficult for human observers to discern, providing a complementary strategy for characterizing longitudinal imaging changes. To the best of our knowledge, inter-fraction changes in routinely acquired MR-Linac images have not been systematically characterized using data-driven approaches.

In this study, we developed an AI model that detects subtle changes in low-field MR-Linac images acquired at short intervals (average two days), adapting a longitudinal image comparison framework [18]. The model achieved over 95% accuracy in determining the temporal order of paired images, outperforming an expert. Temporal ordering provides a self-supervised framework for learning changes throughout radiotherapy. Model logits were associated with treatment progression and biochemical recurrence (BCR). Saliency maps, expert evaluation, and input ablation identified the primary anatomical regions contributing to model predictions and evaluated potential confounding factors. Overall, our study demonstrates the feasibility of detecting subtle changes in routine MR-Linac images and suggests their potential as clinical biomarkers for treatment monitoring and adaptation.

## 2 Material and methods

**Table 1:** Patient characteristics. Continuous variables are reported as median (lower quartile, upper quartile).

Characteristic	Total
**No. of patients**	761
**Age** (years)	73 (67, 77)
**Grade Group** (No.)	
1	20 (3%)
2	250 (33%)
3	98 (13%)
4	27 (4%)
5	63 (8%)
Missing	303 (40%)
**Risk groups** (No.)	
Low	28 (4%)
Intermediate	228 (30%)
High	211 (28%)
Very high	15 (2%)
Missing	279 (37%)
**T Stage** (No.)	
T1	85 (11%)
T2	197 (26%)
T3	51 (7%)
T4	12 (2%)
Missing	416 (55%)
**PSA level** (ng/mL)	
Pre-radiotherapy	
Minimum	3.02 (1.10, 5.74)
Maximum	7.95 (5.27, 14.66)
Missing (No.)	122 (16%)
Post-radiotherapy	
Minimum	0.13 (0.06, 0.51)
Maximum	1.11 (0.30, 3.02)
Missing (No.)	45 (6%)
**BCR** (No.)	
Positive	56 (7%)
Negative	521 (69%)
Missing	184 (24%)
**No. of patients with ADT**	276 (36%)
**No. of fractions**	
5	719 (95%)
Others	42 (5%)
**Days between fractions**	2 (2, 3)

**Table 2:** Performance of the model and the expert.

Pair	Performer	ACC	AUC
$F_{1}$-$F_{L}$	AI	0.95 (0.93, 0.98)	0.99 (0.99, 1.00)
	Expert	0.82 (0.75, 0.87)⁎	–

*All-pairs*	AI	0.91 (0.90, 0.92)	0.97 (0.96, 0.97)
	AI, inference for Sim-$F_{1}$ pairs	0.39 (0.34, 0.45)⁎	0.34 (0.28, 0.40)⁎

* Shows statistically significant differences compared to previous rows (p<.0001).

**Table 3:** Input ablation. Values in the first row are presented as mean ± standard deviation. All other values represent the difference from the baseline mean ± standard deviation. ↓: Performance degradation compared to the baseline indicates strong model dependence on the targeted organ regions. The worst case is highlighted in bold. ↑: Higher performance indicates strong model dependence on the targeted organ regions. The best case is highlighted in bold.

Input	Organ	$F_{1}$-$F_{L}$ model		All-pairs model	
		ACC	AUC	ACC	AUC
Baseline	–	**0.95 ± 0.01**⁎	**0.99 ± 0.00**⁎	**0.91 ± 0.01**⁎	**0.97 ± 0.00**⁎

Organ-	Prostate	−0.03 ± 0.01	−0.01 ± 0.00	−0.01 ± 0.01	−0.00 ± 0.00
masked-	Bladder	−0.02 ± 0.01	−0.01 ± 0.00	−0.04 ± 0.01	−0.02 ± 0.00
MR(↓)	Prostate ∪ Bladder	**−0.05 ± 0.02**	**−0.04 ± 0.01**	**−0.04 ± 0.01**	**−0.03 ± 0.00**

Box-	Prostate	−0.07 ± 0.02	−0.06 ± 0.02	−0.04 ± 0.01	−0.02 ± 0.00
masked-	Bladder	−0.04 ± 0.02	−0.02 ± 0.01	−0.08 ± 0.01	−0.06 ± 0.01
MR(↓)	Prostate ∪ Bladder	**−0.20 ± 0.02**	**−0.19 ± 0.02**	**−0.25 ± 0.01**	**−0.25 ± 0.01**

Only-	Prostate	**−0.03 ± 0.02**	**−0.01 ± 0.01**	−0.10 ± 0.01	−0.08 ± 0.01
organ-	Bladder	−0.09 ± 0.02	−0.06 ± 0.01	−0.14 ± 0.01	−0.12 ± 0.01
MR(↑)	Prostate ∪ Bladder	−0.03 ± 0.02	−0.02 ± 0.01	**−0.08 ± 0.01**	**−0.05 ± 0.00**

Mask	Prostate	**−0.05 ± 0.02**	**−0.04 ± 0.01**	−0.16 ± 0.01	−0.15 ± 0.01
(↑)	Bladder	−0.21 ± 0.03	−0.22 ± 0.03	−0.29 ± 0.01	−0.30 ± 0.01
	Prostate ∪ Bladder	−0.09 ± 0.02	−0.07 ± 0.02	**−0.12 ± 0.01**	**−0.13 ± 0.01**

* All cases are significantly different from the baseline results (p<.0001).

### 2.1 Data

The study is HIPAA-compliant and IRB-approved with a waiver of informed consent for retrospective studies. All data were de-identified prior to analysis. We retrospectively collected 1071 patients who underwent MR-guided radiotherapy for prostate cancer from 2018 to 2025, screened to 761 patients for the study (Details of patient selection are provided in Section S1, with a flow diagram in Figure S2). The images include simulation scans (*Sim* in Fig. 1A) and corresponding treatment fractions ($F_{1}$ to $F_{L}$ in Fig. 1A), with average inter-fraction intervals of two days (Figure S2). All images were acquired using a ViewRay MRIdian MR-Linac system (ViewRay Inc., USA) equipped with a 0.35-T MRI, using True Fast Imaging with Steady-State Free Precession (TRUFI) sequence. The in-plane pixel spacing was $1.5\times 1.5\;{\mathrm{mm}}^{2}$, with a slice thickness of 1.5 or 3 mm (2088 and 1617 of 3705 volumes, respectively; 56% and 44%). Images were automatically cropped to (80, 80, 80) volumes centered on the prostate masks (see Section S2 for the segmentation AI model) while retaining the prostate, adjacent tissues, and organs at risk (OARs). No additional preprocessing was performed to minimize alterations to images.

Data were randomly split on a patient-wise basis into training (60%, *n* = 457), validation (20%, *n* = 152), and test data (20%, *n* = 152). All MR images were cropped to dimensions of (80, 80, 80), centered at the centroid of the prostate mask (see Section S2 for segmentation method).

### 2.2 Model

The overall framework (Fig. 1B) is based on Learning-based Inference of Longitudinal imAge Changes (LILAC) [18], which learns meaningful changes from paired longitudinal images via predicting temporal order. As temporal ordering has demonstrated effectiveness in capturing monotonic changes, we adopted it to identify features consistently changing over the course of treatment. We employed a Siamese 3D convolutional neural network [19], [20], [21] with ResNet-18 [22] architecture. The model outputs logits for binary classification, predicting whether the input pair is in the correct or reversed temporal order. Code is provided in https://github.com/anonymized/mr-linac-ai/will_be_added. Further architectural details of the model are available in Section S3.

For training, MR images from multiple fractions were paired in all possible combinations, yielding 2238 pairs from 457 patients. A curriculum-learning strategy was adopted, where initial training used only first-last fraction image pairs ($F_{1}$*–*$F_{L}$ *pairs)*, which exhibit the largest radiation-induced differences and are therefore easier to learn [23]. The model was further trained using all combinations of image pairs (*All pairs*; *All-pairs model*). More details are in Section S4.

**Fig. 1:**
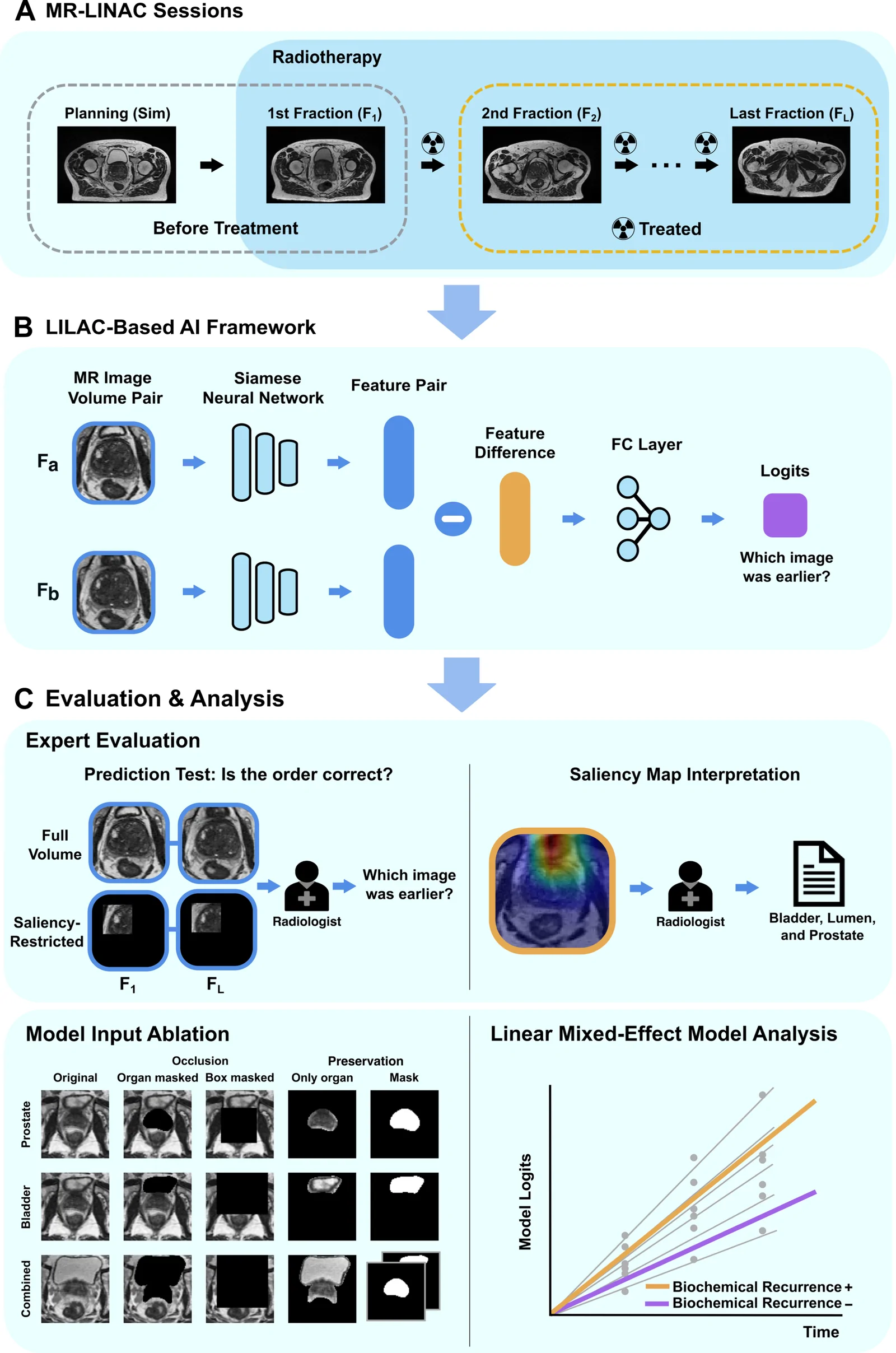
Overview of the MR-Linac workflow and AI framework. (A) Process of MR-Linac sessions. *Sim* refers to the simulation scans obtained prior to treatment sessions. $F_{1}$, $F_{2}$, …, $F_{L}$ represent MR images acquired at the first, second, …, and last fractions, respectively, prior to image-guided radiotherapy. (B) Schematic diagram of the LILAC-based AI model. Paired 3D MR image volumes from fractions are input to a Siamese 3D convolutional neural network with ResNet-18 [22] architecture, in which features of each image are obtained. The differences between these two features are fed into a fully connected (FC) layer. The logits are used for binary classification to predict if the image pairs are in the correct temporal order or not. (C) Experimental design for evaluation and analysis. A radiologist performed the same temporal-ordering task first with full volumes and next with saliency-restricted images. The radiologist also evaluated anatomical regions highlighted by the saliency maps. Model input ablation experiments were designed to evaluate important areas for prediction. Performance changes when the prostate, bladder, or both regions or the surrounding box regions were masked in MR images (Organ Masked, Box Masked) were evaluated, as well as performance for partial MR images encompassing the organ regions (Only Organ). Performance for using only masks (Mask) was evaluated to investigate the effects of the organ shapes, compared to MR intensities. Linear mixed-effects model analysis was performed with biochemical recurrence as a fixed effect to evaluate its association with model logits.

### 2.3 Assessment of changes across fractions via model performance

The held-out test data (152 patients, 732 image pairs) were used to evaluate the performance across all experiments.

#### Temporal ordering performance evaluation.—

Model performance was assessed using accuracy and the area under the receiver operating characteristic curve (AUC), with confidence intervals (CI) obtained from 1000 bootstrap resamples of the test data.

• **Comparison to expert:** A radiologist with over 15 years of experience performed a pairwise image comparison task to determine the correct temporal order of the same $F_{1}$-*-*$F_{L}$ image pairs and assessed treatment changes.
• **Sim-F1 test:** We hypothesized that model performance on Sim (not included in training) and $F_{1}$ pairs would be near random, on the premise that the model primarily captures treatment-related effects. As both images are acquired prior to the radiotherapy session for image guidance purposes, they are not exposed to radiation (Fig. 1A,B).

#### Fraction-pairwise performance evaluation.—

As shown in Kim et al. [21], the model output reflects the magnitude of changes between image pairs. Based on this observation, we examined logits of the *All-pairs* model across fraction levels. To further quantify the relationship between fraction intervals and logits, we computed the Pearson correlation coefficient and conducted a linear mixed-effects model analysis to consider within-patient correlation (See Section S5 for more details).

#### Association between model logits and biochemical recurrence.—

To evaluate the clinical relevance, we analyzed whether the model logits across fractions differed according to BCR using linear mixed-effects models. BCR was defined according to the Phoenix definition [24] as a post-treatment prostate-specific antigen (PSA) increase of 2 ng/mL above the nadir [24]. To test whether the population-level fixed-effect slope differed by BCR group, we compared two linear mixed-effects models using a likelihood ratio test. The full model included separate population-level fixed-effect slopes for the BCR-positive and BCR-negative groups and the reduced model included a single common population-level fixed-effect slope across both groups. Both models included subject-specific random slopes. The association was evaluated after adjusting for established prognostic factors, including baseline PSA, Grade Group, risk group, T stage, and history of androgen deprivation therapy (ADT). See Section S5 for more details.

**Fig. 2:**
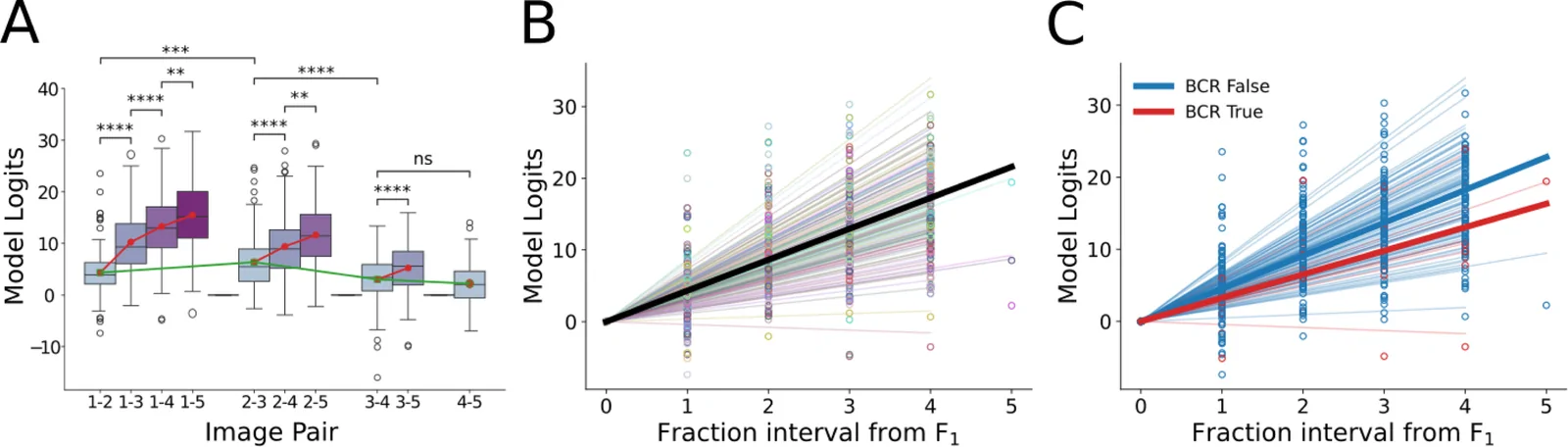
Association between model logits and radiotherapy course. (A) Model logits of the *All-pairs* model for different image pairs. Model logits reflect the model’s confidence in its predictions, which can be interpreted as the magnitude of changes detected between paired images. Pairs of numbers on the x-axis indicate the fractions at which the paired images were acquired. Cases were grouped by the first fraction and ordered and color-coded by the interval between the paired fractions. Stars indicate statistical significance between compared cases (ns: .05<p, **: .001<p≤.01, ***: .0001<p≤.001, ****: p≤.0001). Means within the same first fraction group are shown in red, whereas means with the same fraction interval but different first fraction groups are shown in green. (B) Linear mixed-effects model showing the relationship between fraction intervals from the first fraction and model logits (black), with patient-wise gradients shown in multicolor. (C) Linear mixed-effects model with biochemical occurrence (BCR). Solid lines represent the fixed-effect trajectories estimated by the linear mixed-effects model for patients without BCR (blue) and with BCR (red).

### 2.4 Identification of anatomical regions with changes across fractions using AI model

#### Saliency map.—

Saliency maps highlight regions that the model identifies as contributing most strongly to the differences between a given image pair, which are likely to reflect radiotherapy-induced effects. We used a modified Gradient-weighted Class Activation Mapping (Grad-CAM) [18], [25] to calculate the saliency maps. Implementation details and population-level visualization on an atlas are available in Section S6.

#### Input ablation.—

Input ablation experiments were conducted to identify regions critical for the model’s prediction and assess organ size as a potential confounder, by examining model performance changes depending on input changes (Fig. 1C). Occlusion was performed using either the segmented regions (see Section S2 for segmentation method) themselves (*organ-masked-MR* input) or bounding boxes surrounding the segmented areas (*box-masked-MR* input), where lower performance indicates stronger model reliance on the occluded organ regions. Preservation was performed by retaining only the prostate, bladder, or both segmented regions in the MR images (*only-organ-MR* input), where higher performance indicates stronger model reliance on the preserved organ regions. To further assess the decoupled effect of organ size from intensity, segmented organ masks (prostate, bladder, or both) were used (*mask* input).

#### Expert saliency-restricted MR evaluation.—

The radiologist performed the same temporal-order identification task using images restricted to the regions highlighted by the saliency maps, with the saliency maps themselves not shown (*saliency-restricted-MR*; implementation details in Section S7). The radiologist also documented the rationale for each decision. The overlaid saliency maps were later provided for expert’s evaluation in order to interpret the model’s decision-making process.

### 2.5 Image analysis

Quantitative image analysis was performed to further investigate radiotherapy-induced changes and to compare them with the model and expert evaluations. Changes in organ volume, and in the mean and standard deviation of image intensity for the prostate and bladder were assessed for all patients between $F_{1}$ and $F_{L}$. Prostate and bladder were defined using inferred masks generated by the deep learning model (See Section S2 for details about segmentation).

**Fig. 3:**
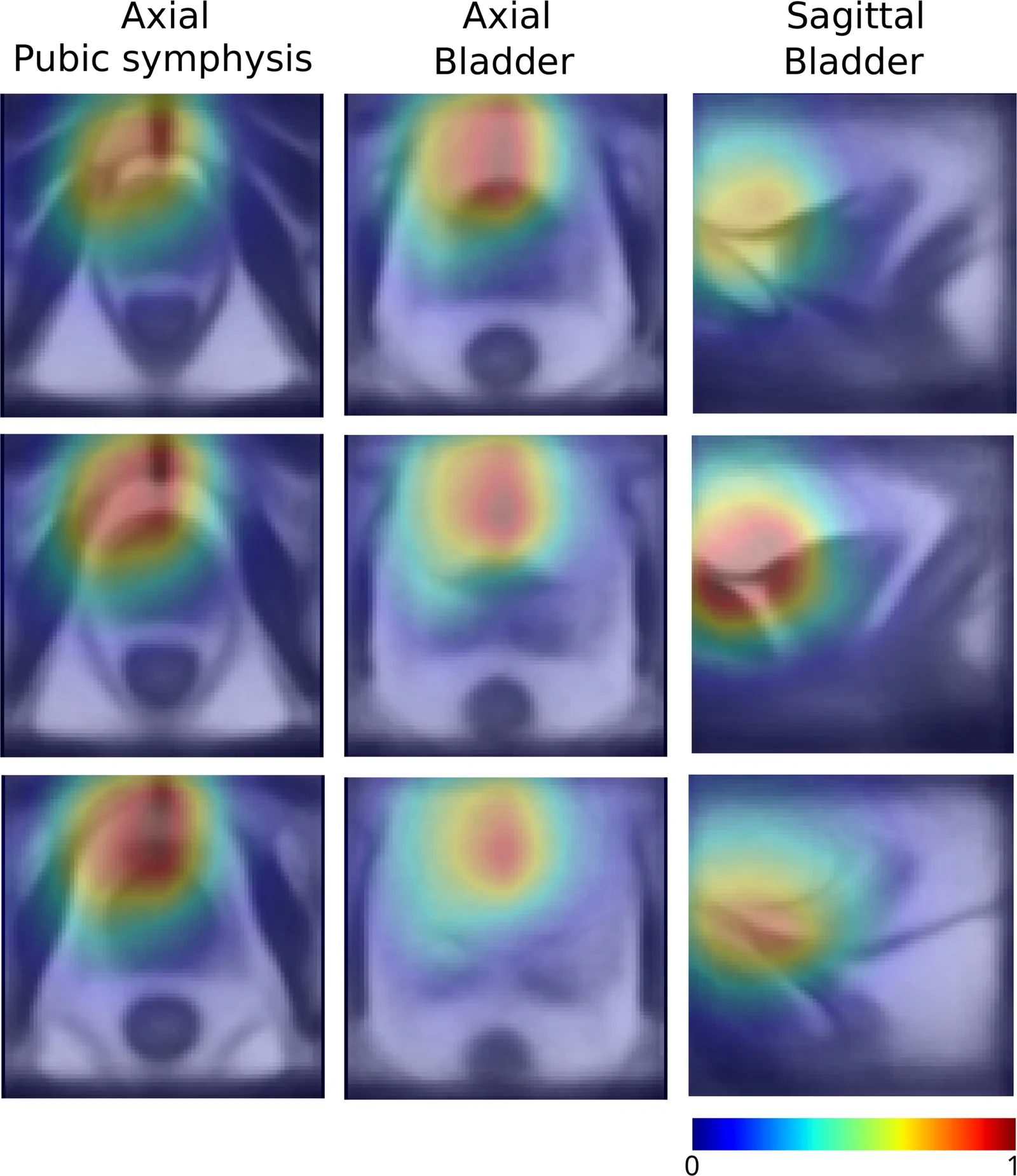
Saliency map on an atlas. The atlas was built from $F_{1}$ of all patients in the test data. The visualized heatmap is the mean of saliency maps for all patients in the test data, obtained from *All-pairs* model inference on $F_{1}$-$F_{L}$ pairs and transformed into the atlas space. Column titles indicate the slice orientations (axial, sagittal) and the primary regions of interest highlighted by the saliency maps, although these regions span multiple organs.

### 2.6 Statistical analysis

Continuous patient characteristics, including age and pre- and post-radiotherapy PSA levels, were summarized as medians and interquartile ranges (IQRs), and differences among the training, validation, and test sets were assessed using the Kruskal–Wallis test. Categorical characteristics, including Grade Group, risk group, T stage, and BCR status, were summarized as counts and compared across the data splits using the chi-square test. An independent two-sided t-test was conducted for comparisons of scores (accuracy and AUC) and patient characteristics. The Wilcoxon signed-rank test was used to compare model logits between the BCR and non-BCR groups, and organ volume and intensity from $F_{1}$ to $F_{L}$. For all statistical tests, p-value of 0.05 was used as the threshold for statistical significance. See Section S8 for details on the tools used.

## 3 Results

### 3.1 Patient characteristics

All patients in the cohort (Table 1, Table S1) were diagnosed with elevated PSA levels and prostate cancer. The median PSA follow-up duration was 921 days (IQR 537–1327; range 2–2800), with a median of 6 PSA measurements per patient (IQR 3–11; range 1–66) and a median interval of 91 days between consecutive measurements (IQR 44–141; range 1–2334). All but 42 patients received five fractions of MR-Linac treatment, mostly administered every two days (Figure S2). Patient characteristics, including age, pre- and post-radiotherapy PSA levels, Grade Group, risk group, T stage, and BCR status, did not differ significantly among the training, validation, and test sets (all p>.05).

All further analyses were performed using the test set (with consistent results obtained using the entire dataset). However, because of missing PSA values, the linear mixed-effects model analysis for BCR was performed using patients with available BCR labels from the entire dataset (n = 577) to maximize the available sample size. Model logits did not differ significantly between patients with available and missing BCR labels (n = 276; p>.05).

### 3.2 Evaluation of changes in images across fractions via model performance

Because temporal order is inferred from image differences, output logits were used as a surrogate measure of change across radiotherapy fractions.

#### Model performance and comparative analysis.—

The model performance on the test set was quantitatively evaluated (Table 2). The $F_{1}$-$F_{L}$ model yielded an accuracy of 0.95 (95% CI: 0.93, 0.98), and the AUC of 0.99 (95% CI: 0.99, 1.00). The model outperformed the expert achieving an accuracy of 0.82 (95% CI: 0.76, 0.88), of which the difference was statistically significant (independent two-sided t-test, p<.0001).

The *All-pairs* model achieved an accuracy of 0.91 (95% CI: 0.90, 0.92) and an AUC of 0.97 (95% CI: 0.96, 0.97). In contrast, inference on Sim-$F_{1}$ pairs yielded a significantly lower accuracy of 0.39 (95% CI: 0.34, 0.45) and AUC of 0.34 (95% CI: 0.28, 0.40) (independent two-sided t-test, p<.0001). The model achieved high accuracy on images acquired during radiotherapy but performed poorly on pre-radiotherapy images (Sim-$F_{1}$), indicating that radiotherapy-associated changes may underlie the model’s decisions. Patients who underwent ADT showed no statistically significant differences in model logits (independent two-sided t-test).

#### Association between model performance and radiotherapy course.—

We hypothesized that image differences between radiotherapy fractions would increase with longer fraction intervals due to potential cumulative radiation exposure, if the detected temporal changes are associated with radiotherapy. To examine this, the performance of the *All-pairs* model for each fraction pair was evaluated (Fig. 2 and Figure S3). Model logits, reflecting the magnitude of detected changes, were positively correlated with the inter-fraction interval (Pearson r=0.59, p<.0001; Fig. 2A), as well as accuracy and AUC all increased proportionally with the fraction intervals (Figure S3). Within groups of image pairs sharing the same first image (e.g. 1–2, 1–3, 1–4, and 1–5), the mean model logits increased with the fraction interval (red lines in Fig. 2A). Among adjacent image pairs (1–2, 2–3, 3–4, and 4–5), the $F_{2}$-$F_{3}$ pairs exhibited significantly higher performance than other one-interval pairs (green lines in Fig. 2A), suggesting that changes are most pronounced between the second and third fractions. These results demonstrate that the model predictions can quantify changes across radiotherapy.

Incorporating individual variability in the linear mixed-effects model resulted in a significant difference from the reduced model without random effects, according to the likelihood ratio test (p<.0001) (Fig. 2B). This indicates that the changes vary across patients, suggesting heterogeneous treatment effects.

To evaluate the clinical relevance, we assessed whether model logits differed by BCR status after treatment using linear mixed-effects models (Fig. 2C). Population-level fixed-effect slopes differed significantly by BCR group, as indicated by a likelihood ratio test comparing models with and without group-specific slopes (p<.05). Notably, the fixed-effect slope was lower in the BCR-positive group, suggesting that patients who later developed BCR exhibited smaller changes in model logits over the course of radiotherapy. The association between BCR status and longitudinal changes in model logits remained significant after adjustment for baseline PSA, Grade Group, risk group, T stage, and ADT history (p<.05).

### 3.3 Localization of changes driving model predictions

#### Saliency map.—

Modified Grad-CAM [21], [25] based saliency maps were used to interpret which regions contribute to the model’s predictions. According to the radiologist’s evaluation, the saliency peaks most frequently occurred in the bladder lumen (20.53%) and pubic symphysis (19.21%), with the prostate commonly involved (Table S2). Group-level visualization of the heatmaps on the atlas of all patients is shown in Fig. 3 (Peak distribution of the heatmaps in Figure S4), of which the primary peak regions are the pubic symphysis and bladder, while the area spans multiple anatomical regions, including the prostate.

#### Input ablation.—

Input ablation experiments were conducted to identify anatomical regions critical to the model’s performance (Table 3). All input ablation test results were significantly different from the baseline result. Occluding both prostate and bladder regions in MR (*Organ-masked-MR* and *Box-masked-MR*) shows a significant drop in the performance, indicating that the prostate, bladder, and surrounding regions contain important radiotherapy-related changes. *Box-masked-MR* caused greater performance degradation than *Organ-masked-MR*, suggesting that shape changes may contribute to the model’s predictions, which motivated the evaluation of *Mask* input.

Preservation experiments show the effect of organ shape and texture as input (*Only-organ-MR*) and only shape as input (*Mask*). Using masks alone (*Mask*) reduced performance relative to *Only-organ-MR*, although accuracy remained above 0.9 ($F_{1}$–$F_{L}$ model) and 0.8 (*All-pairs* model). Prostate mask had a larger impact than bladder mask. The results suggest that prostate size is a major contributor, but intensity also contributes. The performance degradation relative to the baseline was greater for the *All-pairs* model than $F_{1}$–$F_{L}$ model, indicating that shape and texture information are essential to capture subtle inter-fraction changes.

The *All-pairs* model performed best when both prostate and bladder were preserved, whereas the $F_{1}$–$F_{L}$ model achieved higher performance when only prostate was preserved. This indicates that the most pronounced changes between pre- and post-treatment are localized to the prostate, whereas more subtle inter-fraction changes involve both prostate and bladder.

#### Expert saliency-restricted-MR evaluation.—

The expert additionally assessed the model’s saliency maps by performing a prediction test on *saliency-restricted MR* images (Prediction Test on Saliency-Restricted-MR in Fig. 1C). Although the field of view of the images is limited, the expert achieved an accuracy of 0.72 (95% CI: 0.66–0.79), supporting the relevance of the model-highlighted regions for detecting temporal changes.

For the full-volume prediction test, the expert specified the decision criteria in descending order of importance: (1) darkening of the prostate, (2) reduced distinction between the peripheral zone and transition zone borders, (3) enlargement of the prostate, and (4) reduced definition of the prostate’s outer edge. The expert primarily relied on changes in prostate volume and MR image characteristics, including prostate brightness and clarity of the prostate edges. The rationale was similar in the *saliency-restricted-MR* test (Table S3).

### 3.4 Organ volume and intensity changes in images during radiotherapy

Changes in prostate and bladder volume and intensity between $F_{1}$ and $F_{L}$ were analyzed to validate the model and expert findings (Figure S5). Prostate volume increased significantly from $F_{1}$ to $F_{L}$, whereas bladder volume decreased significantly (one-sided Welch’s t-test, p<.0001). Both organs became darker, with the mean intensity in each region decreasing significantly from $F_{1}$ to $F_{L}$ (independent one-sided t-test, p=.02 and .001, respectively). Additionally, the variance of intensity, reflecting heterogeneity, increased significantly in the prostate, whereas it decreased significantly in the bladder (one-sided independent t-test, p=.002 and p<.0001, respectively). Notably, the cropped MR image intensities remained largely stable across fractions (Figure S6), suggesting that systematic scanner scaling drift or calibration changes were unlikely to account for the observed model performance or changes in prostate intensity.

The observed increase in prostate volume and reduction in brightness is consistent with the findings of both the model and the expert assessment. These results suggest that the model’s detection of inter-fractional changes may be partially explained by changes in prostate volume and intensity.

## 4 Discussion

In this study, we demonstrate that AI-based analysis of MR-Linac images can detect subtle inter-fraction changes occurring over short time scales (two days on average) during radiotherapy for prostate cancer. Inter-fraction changes are difficult to identify visually, and previous studies have largely focused on predefined features, such as prostate volume or multiparametric and quantitative MRI biomarkers. Our AI-based approach learns longitudinal imaging features directly from routinely acquired MR-Linac images without requiring predefined imaging biomarkers.

The model achieved near-perfect performance, outperforming expert assessment (Table 2), suggesting the presence of subtle differences that are difficult to detect visually. Its performance on image pairs acquired before the delivery of radiotherapy (Sim-*-*$F_{1}$) was significantly lower than on radiotherapy-affected pairs, suggesting a possible association between the detected changes and radiation exposure. Performance also increased with longer inter-fraction intervals, consistent with cumulative radiation effects (Fig. 2). Failure cases occurred mainly in consecutive-fraction pairs and were not associated with patient characteristics or imaging parameters.

Anatomical regions contributing to the model’s prediction were investigated through saliency maps and input ablation experiments. The saliency maps mainly highlighted the pubic symphysis, bladder, and prostate (Fig. 3, Table S2). Radiotherapy can cause genitourinary toxicities [26], [27], [28], suggesting that the bladder may be affected. In contrast, relatively little has been reported regarding radiation effects on the pubic symphysis. Saliency maps identify image regions that contribute to the model’s prediction but do not establish underlying biological mechanisms. The prominence of the pubic symphysis may therefore reflect its role as a source of contextual image information. Further studies are required to determine the biological significance, if any, of this finding.

Quantitative analysis confirmed that the prostate enlarged and darkened with increased intensity heterogeneity (Figure S5), which is consistent with the radiologist’s evaluation (Table S3) and previous reports of transient prostate swelling during radiotherapy. The bladder decreased in volume and signal intensity, and its signal became more uniform (Figure S5). Radiotherapy may have contributed to prostate enlargement and associated bladder compression. However, the observed decrease in bladder volume should be interpreted cautiously because bladder filling is influenced by physiological factors such as hydration status and compliance with bladder preparation protocols. Signal darkening occurred in both, but increased intensity variability was observed only in the prostate, suggesting different responses from targeted versus non-targeted radiation.

Potential confounders include scanner drift and changes in bladder volume, in addition to changes occurring during radiotherapy. We show that image intensities remained stable across fractions (Figure S6), suggesting that scanner drift was unlikely to explain the model performance and observed intensity changes in prostate. Nevertheless, some variability in non-quantitative TRUFI signal intensities may remain due to the lack of intensity normalization. Input ablation experiments showed that the model performed poorly when using the bladder mask alone, indicating that bladder size alone is not a primary driver of the model performance.

Temporal ordering was used for self-supervised learning, not directly intended as a clinical application. However, other results and the observed association between model logits and BCR (Fig. 2C) suggest the potential clinical relevance of the model-derived longitudinal imaging changes. Future work should investigate whether these representations can improve prediction of treatment response and clinical outcomes.

Several limitations of this study should be acknowledged. The use of 1.5- (56%) and 3-mm (44%) slice thicknesses resulted in different physical coverage for the fixed-size model inputs, which may have introduced variability in the anatomical context available to the model. This proof-of-concept used data from a single platform, and performance on other platforms remains to be determined. Our findings suggest a possible association between the detected imaging changes and radiotherapy. However, further investigation is needed to determine whether these reflect radiation-specific biological effects.

In conclusion, we show that AI can detect changes in MR-Linac images across radiotherapy for prostate cancer. Results support the feasibility of AI-based identification of in-treatment imaging changes, which may serve as potential imaging biomarkers of treatment response or adverse effects. AI-derived imaging features may support treatment monitoring, adaptation, and personalized radiotherapy.

## CRediT authorship contribution statement

**Seungbin Park:** Writing – review & editing, Writing – original draft, Visualization, Validation, Methodology, Investigation, Formal analysis, Data curation, Conceptualization. **Peilin Wang:** Writing – review & editing, Visualization, Validation, Methodology, Investigation, Formal analysis, Data curation. **Joshua Paik:** Writing – review & editing, Visualization, Validation, Software, Methodology, Formal analysis, Data curation. **Ryan Pennell:** Writing – review & editing, Software, Resources, Methodology, Investigation, Data curation, Conceptualization. **Francesca Khani:** Writing – review & editing, Visualization, Supervision, Software, Resources, Methodology, Investigation, Formal analysis, Data curation, Conceptualization. **Himanshu Nagar:** Writing – review & editing, Methodology, Investigation, Conceptualization. **Timothy McClure:** Writing – review & editing, Methodology, Investigation, Data curation, Conceptualization. **Emily S. Weg:** Writing – review & editing, Supervision, Software, Resources, Methodology, Investigation, Data curation, Conceptualization. **Mert R. Sabuncu:** Writing – review & editing, Supervision, Software, Resources, Project administration, Methodology, Investigation, Funding acquisition, Conceptualization. **Daniel Margolis:** Writing – review & editing, Validation, Supervision, Resources, Methodology, Investigation, Formal analysis, Data curation, Conceptualization. **Heejong Kim:** Writing – review & editing, Visualization, Validation, Supervision, Software, Resources, Project administration, Methodology, Investigation, Funding acquisition, Formal analysis, Data curation, Conceptualization.

## Declaration of generative AI and AI-assisted technologies in the manuscript preparation process

During the preparation of this work, the authors used ChatGPT to identify and correct grammatical errors. The author(s) reviewed and edited the output as needed and take full responsibility for the content of the published article.

## Funding

This work was partly supported by the National Institutes of Health grants K25CA283145, R01AG053949, R01AG064027, and R01AG070988.

## Declaration of competing interest

The authors declare the following financial interests/personal relationships which may be considered as potential competing interests: Seungbin Park reports financial support, administrative support, article publishing charges, equipment, drugs, or supplies, and statistical analysis were provided by National Institutes of Health. Heejong Kim reports financial support, administrative support, article publishing charges, equipment, drugs, or supplies, and travel were provided by National Institutes of Health. Mert R. Sabuncu reports financial support, administrative support, article publishing charges, equipment, drugs, or supplies, and travel were provided by National Institutes of Health. Daniel Margolis reports a relationship with Stratagem Inc that includes: non-financial support. Daniel Margolis reports a relationship with Guerbet that includes: consulting or advisory. Himanshu Nagar reports a relationship with Boston Scientific Corporation that includes: consulting or advisory. Himanshu Nagar reports a relationship with Lantheus that includes: consulting or advisory. Himanshu Nagar reports a relationship with Elekta that includes: consulting or advisory. Mert R. Sabuncu reports a relationship with Cleerly Inc that includes: consulting or advisory and equity or stocks. Mert R. Sabuncu reports a relationship with Medara Health that includes: consulting or advisory and equity or stocks. Given their role as the first author, Seungbin Park had no involvement in the peer-review of this article and has no access to information regarding its peer-review. Given their role as a corresponding author, Heejong Kim had no involvement in the peer-review of this article and has no access to information regarding its peer-review. Given their role as co-authors, Peilin Wang, Joshua Paik, Ryan Pennell, Francesca Khani, Himanshu Nagar, Timothy McClure, Emily S. Seg, Mert R. Sabuncu, and Daniel Margolis, had no involvement in the peer-review of this article and have no access to information regarding its peer-review. Full responsibility for the editorial process for this article was delegated to another journal editor. If there are other authors, they declare that they have no known competing financial interests or personal relationships that could have appeared to influence the work reported in this paper.

## Appendix A Supplementary data

MMC S1 Supplementary sections, tables, and figures. The supplementary materials are cited as S-number labels in the manuscript. MMC S1
