# Supplementary material for: AI-based detection of temporal changes in MR-Linac images acquired during routine prostate radiotherapy
Source: Phys Imaging Radiat Oncol. 2026 Sep 23;41:101081. doi: 10.1016/j.phro.2026.101081 (PMC13634774; doi:10.1016/j.phro.2026.101081)
Supplement: Supplementary file 1 [file mmc1.pdf]

Supplemental Material

S1 Patient screening

Of the 1,071 patients, those who underwent multiple treatment sets ($n = 57$) and those without a prostate or prostate mask ($n = 253$) were excluded (Figure S1), as their imaging characteristics may differ from the rest of the cohort.

S2 Segmentation of Organs in MR Images from MR-Linac Fractions

The prostate, bladder, and rectum are routinely contoured in radiotherapy guide images obtained before treatment by radiation oncologists for treatment planning purposes. Since contouring is routinely performed only on the guide images, we trained an nnU-Net [29] on the guide images to get prostate, bladder, and rectum masks in MR images from MR-Linac fractions. We assumed that the model would generalize to images from fractions, as both were obtained from the same MR-Linac system and target the same anatomical regions with consistent imaging protocols.

The segmentation model was trained, validated, and tested using split datasets of guide MR images with corresponding prostate, bladder, and rectum masks delineated by radiation oncologists. On the test set, the Dice scores of the model were 0.85, 0.95, and 0.82 for prostate, bladder, and rectum. The trained model was then applied to MR images acquired during MR-Linac treatment fractions to generate organ masks. Partial qualitative evaluation of the resulting masks, together with quantitative comparison between the trained MR-Linac temporal-ordering models, demonstrated that this approach outperformed masks obtained via image registration.

We used dilated masks for input ablation experiments, except for the mask-only condition, to ensure that organ boundaries were included in the occluded or preserved regions. Binary masks were morphologically dilated by two voxels in all directions using a 3D 26-connected structuring element.

S3 Model Architecture Details

The overall framework (Figure 1B) is based on learning-based inference of longitudinal image changes (LILAC) [18], which extracts biologically meaningful temporal changes directly from paired longitudinal images. It has shown effectiveness across multiple longitudinal imaging applications, including embryo development, wound healing, brain aging, and Alzheimer's disease. Adapting the LILAC framework, we employed a convolutional neural network (ResNet-18 [22]) with a Siamese architecture [19–21], which takes a pair of images as input and extracts the corresponding feature representations to predict the correct order of the given longitudinal image pair. For Siamese architecture, ResNet-18 [22] with 3D convolutional layers was used, which demonstrated superior performance compared to simple convolutional neural networks (Table S4). The difference between these feature vectors is subsequently fed to a bias-free fully connected layer, enabling the model to learn differences between the two images. The resulting logits are used for binary classification to determine whether the input pair is in the correct or reversed temporal order using the binary cross-entropy loss function.

S4 Training Details

For the temporal-ordering task on image pairs, MR images from fractions were paired in all possible permutations with repetition. Consequently, n^2 image pairs were generated per patient (n: the number of fractions for the patient). In total, this pairing yielded $2,238$ pairs for the training data, 737 pairs for the validation data, and 732 pairs for the test data. Ground truth labels were assigned as 1 for the temporally ordered, 0 for the temporally reversed, and 0.5 for the identical pairs.

A curriculum learning approach was adopted as part of the training strategy [23], where a model is gradually exposed to examples in an order from easy to hard to improve learning efficiency and performance. The approach resulted in improved performance compared with training from scratch (Table S4).

Models were trained for 100 epochs, with the optimal epoch identified as the one yielding the smallest validation loss. Training and validation losses were monitored to check for convergence and overfitting. Model parameters were optimized using the Adam optimizer [30] to minimize the binary cross-entropy loss between the model outputs and the ground truth. The learning rate was

initially set to 0.001 and decayed by a factor of 0.5 every 20 epochs. Training was conducted using the *Python PyTorch* library on a NVIDIA A40 GPU.

S5 Linear Mixed-Effects Model

The association between positive fraction intervals from the first fraction (intervals of F_1-F_X, $1 < X < 7$) and model logits from *All-pairs* model was evaluated using a linear mixed-effects model, with model logits as the dependent variable, fraction intervals as the independent variable, and subjects as the random effect. Because there was no change when comparing the initial time point to itself, the model included no fixed and random intercepts. Model logits were modeled as

$$y_{ij} = (\beta_1 + b_{1i})x_{ij} + \epsilon_{ij}, \tag{1}$$

where y_{ij} represents the model logit for observation j from patient i, x_{ij} represents the fraction interval, β_1 is the population-level fixed-effect slope, b_{1i} is the patient-specific random slope, and ϵ_{ij} is the residual error. To assess the significance of inter-patient variability in the longitudinal relationship, the mixed-effects model was compared with a reduced ordinary least-squares model, $y_{ij} = \beta_1 x_{ij} + \epsilon_{ij}$, using a likelihood ratio test.

To evaluate the clinical relevance, we evaluated whether longitudinal model logits across fraction intervals differed according to biochemical recurrence (BCR) using linear mixed-effects models. BCR was defined from post-treatment prostate-specific antigen (PSA) levels according to the Phoenix definition, with BCR defined as a PSA rise of at least 2 ng/mL above the post-treatment PSA nadir [24], and no BCR otherwise. The PSA nadir was tracked longitudinally from the first post-radiotherapy measurement and updated whenever a lower PSA value was observed. BCR was identified at the first subsequent PSA measurement reaching or exceeding the current nadir plus 2 ng/mL. The full model was defined as

$$y_{ij} = (\beta_{g_i} + b_{1i})x_{ij} + \epsilon_{ij}, \tag{2}$$

where y_{ij} is the model logit for observation j from patient i, x_{ij} is the fraction interval, β_{g_i} is the population-level fixed-effect slope specific to the BCR group (g_i), b_{1i} is the patient-specific random

slope, and ϵ_{ij} is the residual error. The intercept was constrained to zero. The reduced model assumed a common fixed-effect slope across BCR groups, following equation 1, but only using samples with BCR defined. The significance of the BCR-specific longitudinal effect was evaluated using a likelihood ratio test comparing the full and reduced models.

The association was further evaluated after adjustment for established prognostic factors, including baseline PSA, Grade Group, risk group, T stage, and history of androgen deprivation therapy (ADT). Baseline PSA was defined as the maximum PSA value before radiotherapy and categorized as < 1, 1–3, or > 3 ng/mL. Due to missing data for some prognostic factors, this analysis was performed using all available patients across the training, validation, and test sets. Complete data for BCR status and all prognostic factors were available for 81 of 761 patients, who were included in the analysis. The adjusted full model included BCR and all prognostic factors as fixed effects on the fraction-interval slope and was defined as

$$y_{ij} = (\beta_{g_i} + \beta_{psa_i} + \beta_{adt_i} + \beta_{grade_i} + \beta_{risk_i} + \beta_{t_i} + b_{1i})x_{ij} + \epsilon_{ij}, \tag{3}$$

where g_i, psa_i, adt_i, $grade_i$, $risk_i$, and t_i represent the BCR, baseline PSA, ADT, Grade Group, risk group, and T stage categories for patient i, respectively, and the corresponding β terms represent their fixed-effect contributions to the fraction-interval slope. The adjusted reduced model excluded the BCR-specific effect and was defined as

$$y_{ij} = (\beta_{psa_i} + \beta_{adt_i} + \beta_{grade_i} + \beta_{risk_i} + \beta_{t_i} + b_{1i})x_{ij} + \epsilon_{ij}. \tag{4}$$

Both models included patient-specific random slopes and no fixed or random intercepts. A likelihood ratio test was used to determine whether inclusion of the BCR-specific effect significantly improved model fit after accounting for the prognostic factors.

S6 Grad-CAM

A modified Gradient-weighted Class Activation Mapping (Grad-CAM) [18, 25] was calculated to obtain the saliency maps, which visualize the areas in inputs on which the model largely depends for prediction. Gradients of the model outputs were computed with respect to the final activation

maps, and importance was assigned at each spatial location individually. The activation maps were then weighted element-wise by the corresponding gradients to produce the saliency maps. Regions with high Grad-CAM values were analyzed to identify areas likely affected by MR-Linac radiotherapy.

For group-level visualization of the saliency maps, F_1 atlas was constructed from all patients in the test data using *ants.registration.build_template* module in *ANTsPy* [31], python implementation of Advanced Normalization Tools (ANTs). The module begins with an initial template, which is the average of all images. All images were nonlinearly registered using SyN (Symmetric Normalization)—ANTs' diffeomorphic registration model—without rigid or affine alignment, over three iterations. At each iteration, warped images were averaged and blended with the previous template using a blending weight of 0.75 to ensure stable convergence, with deformation updates controlled by a gradient step of 0.2, yielding an unbiased diffeomorphic mean representation of the cohort. Using the constructed atlas, patient-wise saliency maps were transformed into the atlas space, averaged across images, and overlaid for visualization (Figure 3).

S7 Saliency-Restricted-MR Details

Binary masks were generated by thresholding the min-max scaled saliency maps at 0.3. Using these masks, we created bounding boxes that encompassed the thresholded regions. The resulting cropped images were provided to experts for the *saliency-restricted-MR* evaluation.

S8 Statistical Analysis Details

An independent two-sided t-test (*Python scipy.stats.ttest_ind*) was conducted for pairwise comparisons of scores (accuracy and AUC). The Kruskal–Wallis test (*Python scipy.stats.kruskal*) was used to compare continuous patient characteristics, including age and pre- and post-radiotherapy PSA levels, across the training, validation, and test sets. The chi-square test (*Python scipy.stats.chi2 _contingency*) was used to compare categorical patient characteristics, including Grade Group, risk group, T stage, and BCR status, across the data splits. The Wilcoxon signed-rank test (*Python scipy.stats.wilcoxon*) was used to compare model logits between the BCR and non-BCR groups and compare organ volume and intensity from F_1 to F_L.

Table S1: **Patient Characteristics by Dataset Split.** Continuous variables are reported as median (IQR).

Characteristic	Training	Validation	Test
**No. of patients**	457 (60%)	152 (20%)	152 (20%)
**Age** (years)	73 (67, 77)	73 (66, 77)	72.5 (66, 76.25)
**Grade Group** (No.)			
1	11 (2%)	4 (3%)	5 (3%)
2	155 (34%)	49 (32%)	46 (30%)
3	63 (14%)	20 (13%)	15 (10%)
4	21 (5%)	1 (1%)	5 (3%)
5	36 (8%)	15 (10%)	12 (8%)
Missing	171 (37%)	63 (41%)	69 (45%)
**Risk groups** (No.)			
Low	10 (2%)	9 (6%)	9 (6%)
Intermediate	138 (30%)	43 (28%)	47 (31%)
High	128 (28%)	37 (24%)	46 (30%)
Very high	8 (2%)	6 (4%)	1 (1%)
Missing	173 (38%)	57 (38%)	49 (32%)
**T Stage** (No.)			
T1	47 (10%)	16 (11%)	22 (14%)
T2	124 (27%)	33 (22%)	40 (26%)
T3	32 (7%)	9 (6%)	10 (7%)
T4	9 (2%)	2 (1%)	1 (1%)
Missing	245 (54%)	92 (61%)	79 (52%)
**PSA level** (ng/mL)			
Pre-treatment			
Minimum	3.33 (1.16, 6.06)	2.55 (0.75, 5.00)	2.98 (1.22, 5.43)
Maximum	8.45 (5.40, 14.24)	7.47 (5.02, 15.71)	7.33 (4.84, 15.46)
Missing	70 (15%)	27 (18%)	25 (16%)
Post-treatment			
Minimum	0.11 (0.06, 0.49)	0.13 (0.06, 0.40)	0.20 (0.06, 0.68)
Maximum	1.11 (0.34, 3.16)	0.93 (0.28, 2.44)	1.30 (0.25, 2.90)
Missing	29 (6%)	5 (3%)	11 (7%)
**BCR** (No.)			
Positive	35 (8%)	10 (7%)	11 (7%)
Negative	311 (68%)	114 (75%)	96 (63%)
Missing	111 (24%)	28 (18%)	45 (30%)
**No. of patients with ADT**	161 (35%)	64 (42%)	51 (34%)
**No. of fractions**			
5	436 (95%)	143 (94%)	140 (92%)
Others	21	9	12
**Days between fractions**	2 (2, 3)	2 (2, 3)	2 (2, 3)

Index	Expert Evaluation of Grad-CAM Peak	# of Cases
1	Bladder lumen	31 (20.53%)
2	Pubic symphysis	29 (19.21%)
3	Bladder lumen, public symphysis, and periprostatic fascia (1 + 2 + 8)	18 (11.92%)
4	Pubic symphysis and bladder lumen (1 + 2)	17 (11.26%)
5	Base of prostate at urethral inlet	8 (5.30%)
6	Public symphysis and base of prostate at urethral inet (2 + 5)	8 (5.30%)
7	Pubic symphysis and periprostatic fascia (2 + 8)	7 (4.64%)
8	Periprostatic fascia	6 (3.97%)
9	Bladder wall	5 (3.31%)
10	Bladder lumen and lateral prostate (1 + 14)	4 (2.65%)
11	Random fascia above the bladder	4 (2.65%)
12	Urethral inlet and periprostatic fascia (5 + 8)	3 (1.99%)
13	Bladder apex	2 (1.32%)

Table S2: **Expert evaluation of peak regions of saliency maps from the *All-pairs* model on F_1-F_L pairs.** Regions with only one case are omitted from the table: prostate peripheral zone, pubic symphisis and lateral prostate possibly peripheral zone (2 + 14), anterior prostate and possibly artifact, pubic symphysis and tissue above bladder (2 + other), urethral inlet and bladder lumen (1 + 5), like 3 but ill-defined, ill-defined areas spanning the pubic symphysis and prostate base with additional bilateral areas in fascia above the bladder, periprostatic fascia and fascia above the bladder (8 + 11), and bladder lumen and periprostatic fascia (1 + 8).

**Expert Rationale**	**# of Cases**
Areas in the PZ get darker.	85 (56%)
Areas in the TZ get darker.	51 (34%)
TZ features get indistinct.	13 (9%)
Prostate missing but MR signals get darker	2 (1%)

Table S3: **Expert rationales for temporal ordering decisions on *saliency-restricted-MR* images.**

Pair	Model & Training Scheme	ACC	AUC
F_1-F_L	CNN-3D	0.89	0.96
All	CNN-3D, Curriculum learning from the F_1-F_L model	0.89	0.96
	CNN-3D, From scratch	0.88	0.95
	ResNet-18-3D, From scratch	0.88	0.95

Table S4: **Performance of various models and training schemes.** CNN-3D consists of four blocks of 3D convolutional layer (number of output channels set as 16, 32, 64, 16, respectively), batch normalization, leaky ReLU, and average pooling. The ResNet-18-3D model trained with curriculum learning, which was used in the study for subsequent analyses, demonstrated superior performance compared with the CNN-3D and the ResNet-18-3D model trained from scratch.

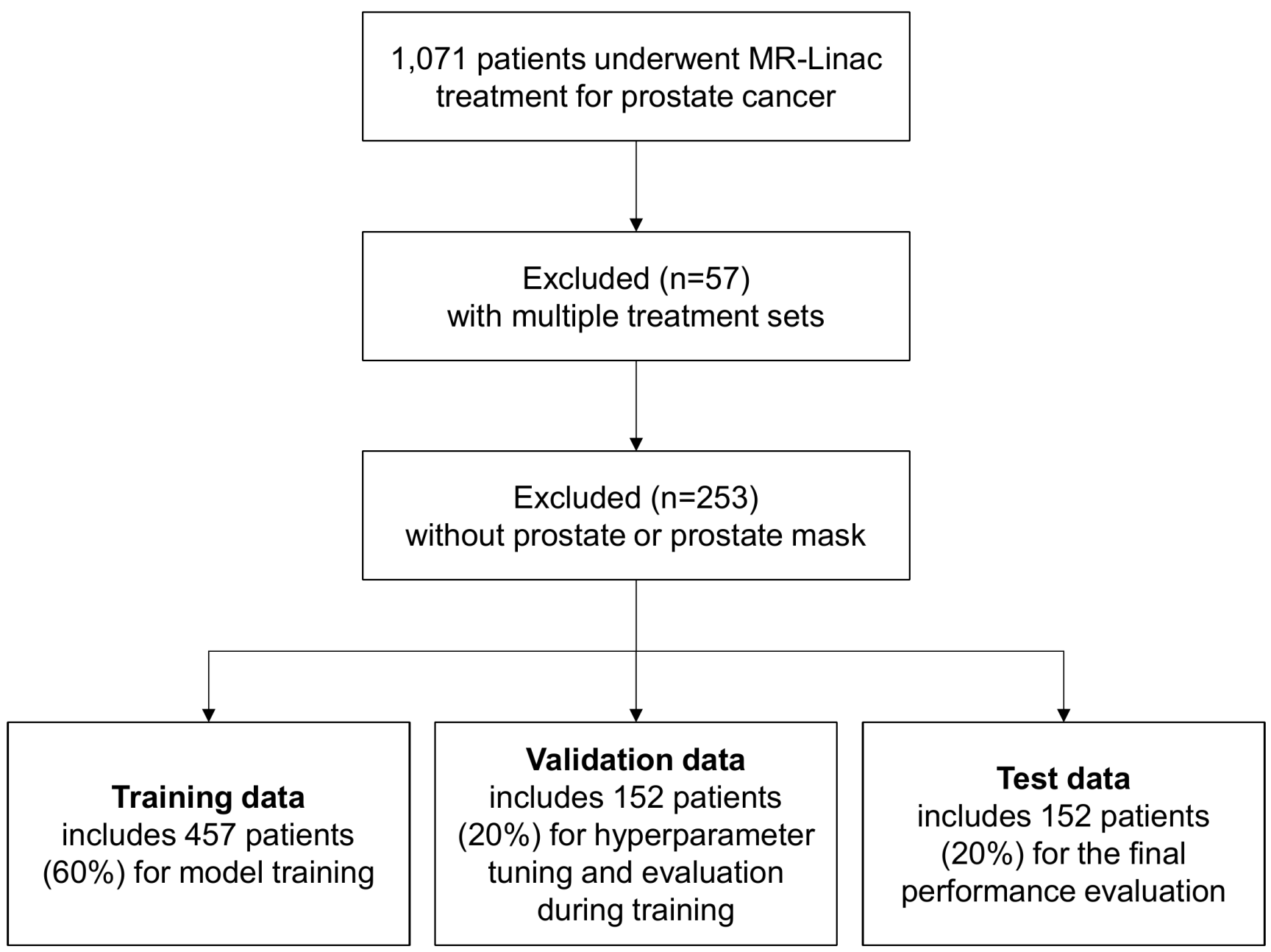

Figure S1: **Flow diagram of cases, indicating inclusion and exclusion of patients, and random split of the data.**

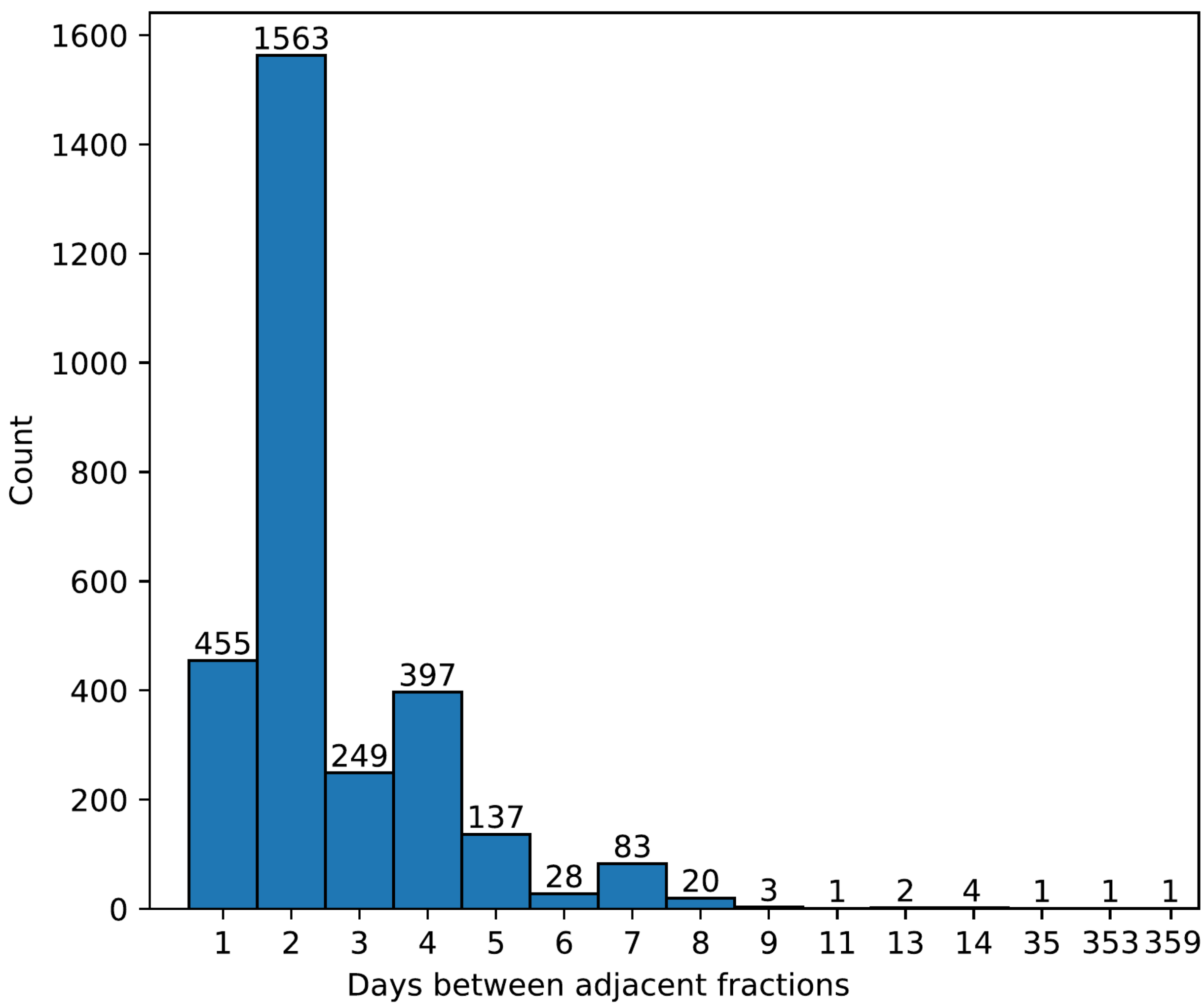

Figure S2: **Histogram of days between adjacent fractions.**

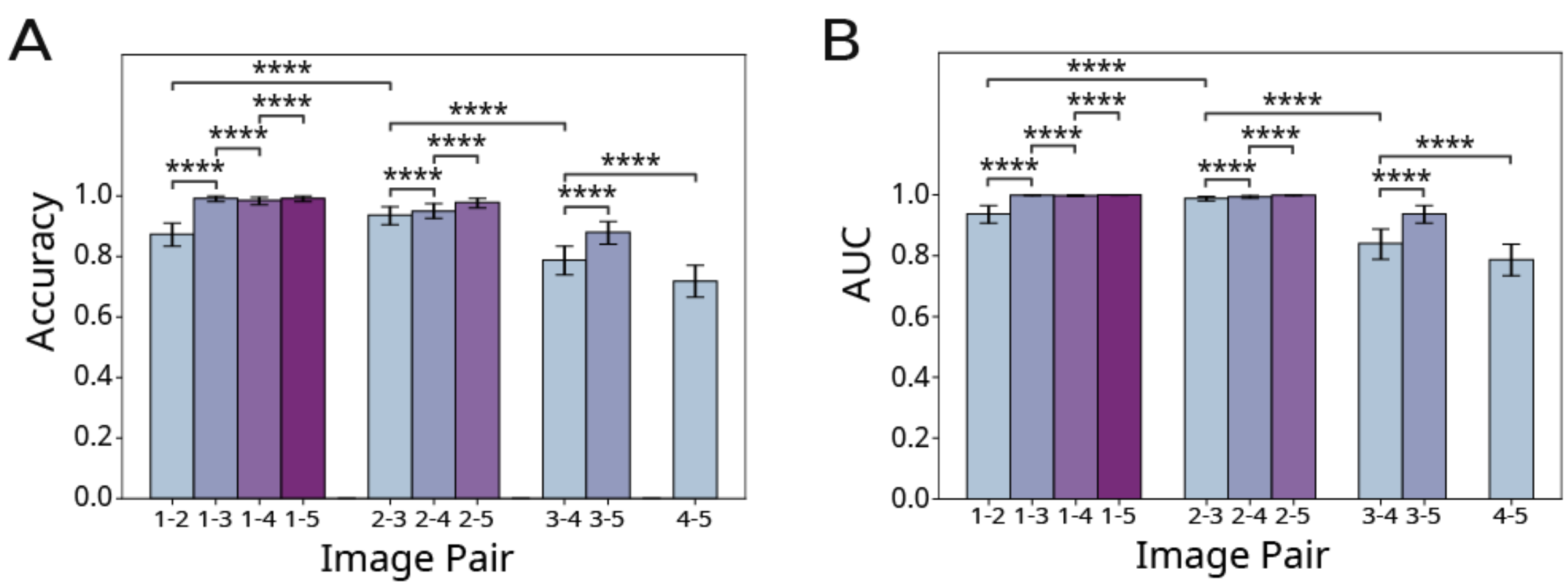

Figure S3: **Pairwise performance of the *All-pairs* model.**(A) Accuracy and (B) AUC of the *All-pairs* model for different image pairs. Pairs of numbers on the x-axes indicate the fractions at which the paired images were acquired. Cases were grouped by the first fraction and ordered and color-coded by the interval between the paired fractions. Stars indicate statistical significance between compared cases (independent two-sided t-test; ****: $p \leq .0001$).

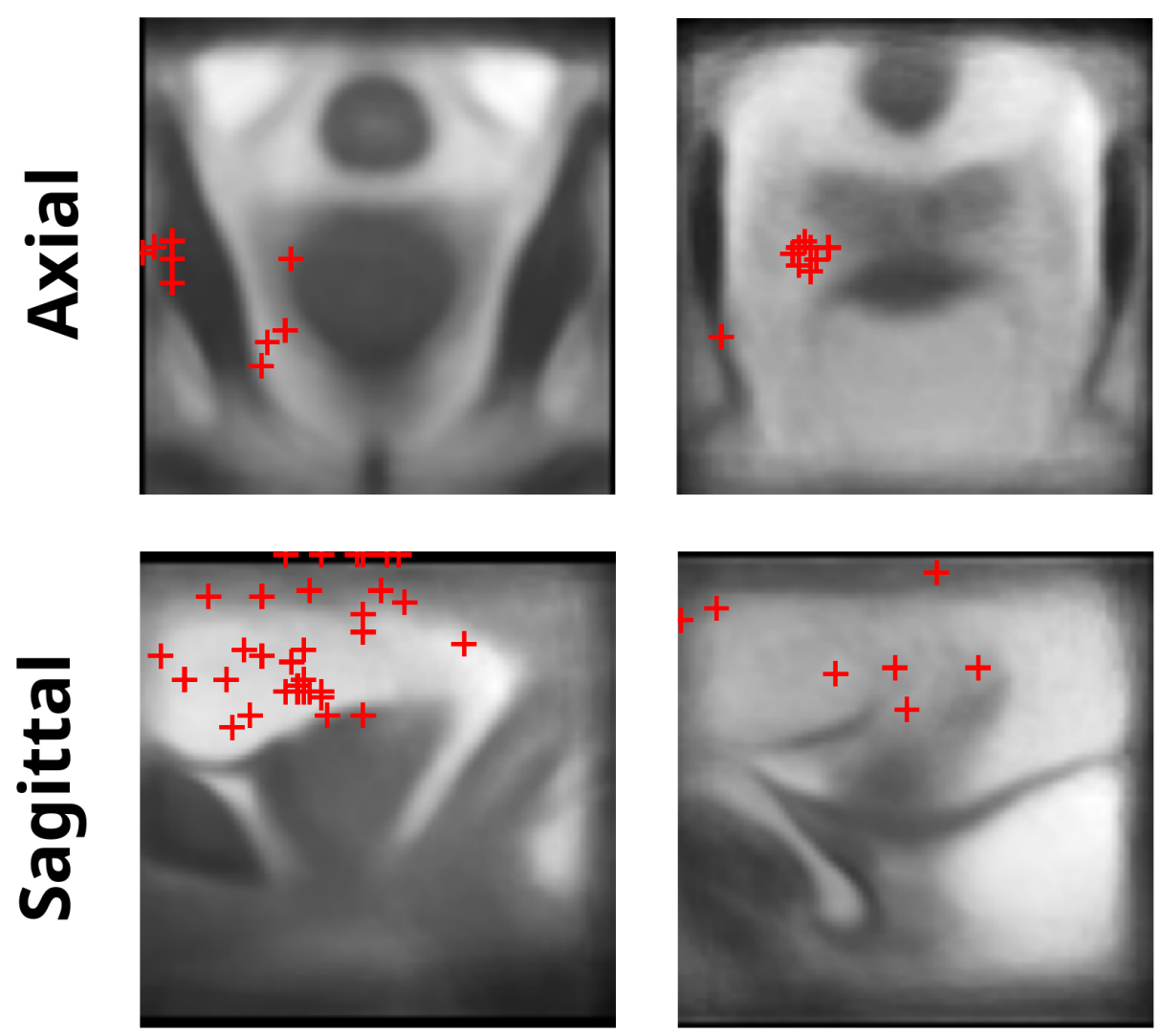

Figure S4: **Peaks of saliency maps of patients in the test data on MR image atlas.**

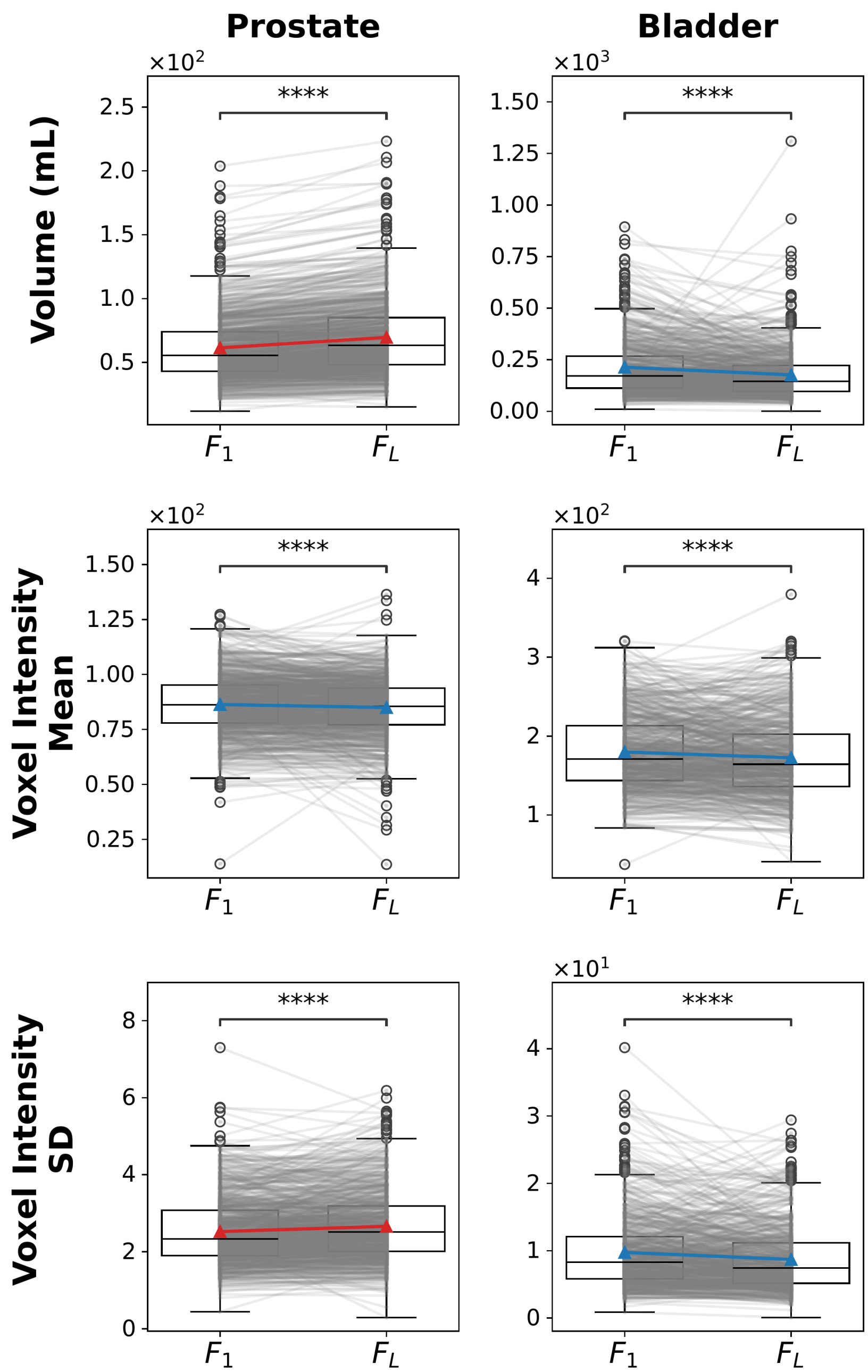

Figure S5: **Changes in volume and intensity of prostate and bladder in full MR images.** An increase in the difference between the two means is indicated in red, whereas a decrease is indicated in blue. Stars indicate statistical significance of the difference (Wilcoxon signed-rank test; ****: $p \leq .0001$)

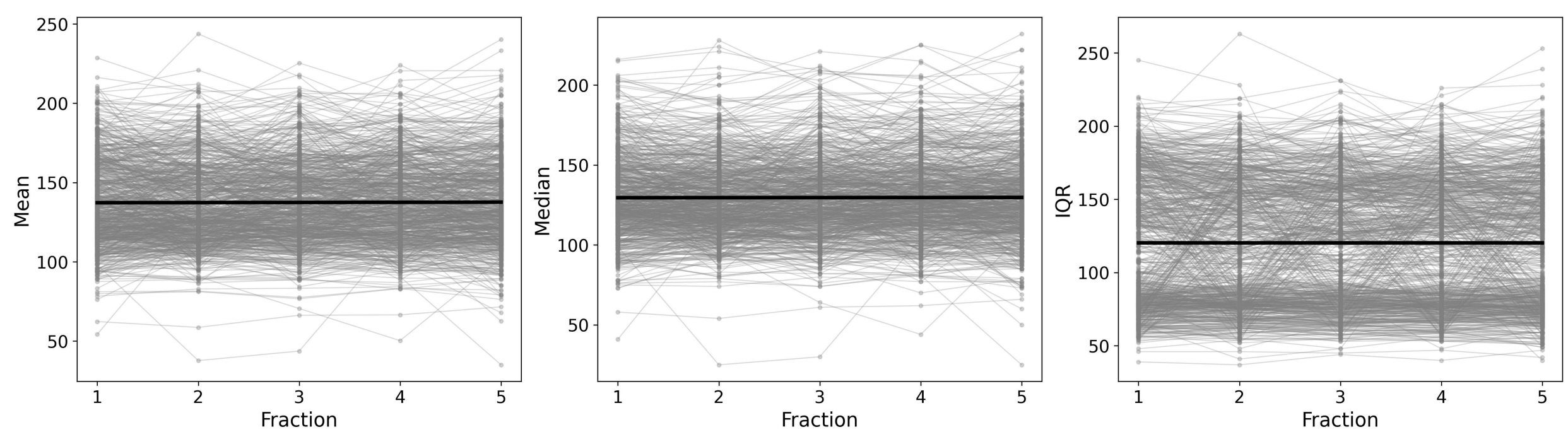

Figure S6: **Stability of cropped MR image intensity statistics across treatment fractions.** Mean (left), median (center), and interquartile range (IQR; right) of voxel intensities for the cropped MR images across the five treatment fractions. Each gray line represents an individual patient trajectory. The solid black line represents the population-level trend estimated from a linear mixed-effects model with treatment fraction as a fixed effect and patient-specific random intercepts and slopes. No obvious systematic global intensity shift was observed across treatment fractions. The estimated slopes for the mean, median, and IQR were 0.64, 0.81, and 0.99, respectively (all $p > .05$)
